# Develop the artificial neural network approach to predict thermal transport analysis of nanofluid inside a porous enclosure

**DOI:** 10.1038/s41598-023-48412-x

**Published:** 2023-11-29

**Authors:** Saleem Nasir, Abdallah S. Berrouk, Taza Gul, Aatif Ali

**Affiliations:** 1 Mechanical Engineering Department, Khalifa University of Science and Technology, P.O. Box 127788, Abu Dhabi, United Arab Emirates; 2 Center for Catalysis and Separation (CeCaS), Khalifa University of Science and Technology, P.O. Box 127788, Abu Dhabi, United Arab Emirates; 3 Department of Mathematics, City University of Science and Information Technology, Peshawar, 25000 Pakistan; 4 School of Mathematical Sciences, Jiangsu University, Zhenjiang, 212013 Jiangsu China

**Keywords:** Engineering, Mathematics and computing

## Abstract

This study explores the impacts of heat transportation on hybrid (Ag + MgO) nanofluid flow in a porous cavity using artificial neural networks (Bayesian regularization approach (BRT-ANN) neural networks technique). The cavity considered in this analysis is a semicircular shape with a heated and a cooled wall. The dynamics of flow and energy transmission in the cavity are influenced by various features such as the effect of magnetize field, porosity and volume fraction of nanoparticles. To explore the outcomes of these features on hybrid nanofluid thermal and flow transport, a BRT-ANN model is developed. The ANN model is trained using a dataset generated through numerical scheme. The trained ANN model is then used to predict the heat and flow transport characteristics for various input parameters. The accuracy of the ANN simulation is confirmed through comparison of the predicted results with the results obtained through numerical simulations. By maintaining the corrugated wall uniformly heated, we inspected the levels of isotherms, streamlines and heat transfer distribution. A graphical illustration highlights the characteristics of the Hartmann and Rayleigh numbers, permeability component in porous material, drag force and rate of energy transport. According to the percentage analysis, nanofluids (Ag + MgO/H_2_O) are prominent to enhance the thermal distribution of traditional fluids. The study demonstrates the potential of ANNs in predicting the impacts of various factors on hybrid nanofluid flow and heat transport, which can be useful in designing and optimizing heat transfer systems.

## Introduction

Improving the energy transmission rate of traditional base fluids is the primary issue faced by the contemporary disciplines of engineering and science. To promote the thermodynamic efficiency along with cooling processes, like energy transmission, cooling of electronics components and vehicle coolant with the highest thermal efficiency, the reduction of heating and the implementation of the accurate procedure for achieving increased constancy. Researchers and academics were therefore fascinated to examine why suspending solid' atoms transferred energy as compared to more ordinary working liquids. Maxwell^1^ initially endeavored to improve the rate of heat transmission of common fluids by incorporating tiny particles. Following extensive research, Choi^2^ concluded that a particular kind of nano-sized particle dispersing, also known as a nanofluid, can be added to a base liquid to increase thermal efficiency. As a result of the discovery of this novel idea, scientists are now extremely interested in exploring the applications of nanofluids. A comprehensive parametric simulation was used by Wakif et al.^3^ to investigate various sophisticated applications of nanofluids. With the help of nanofluid flow, the heat exchange was improved in the study of Elnaqeeb et al.^4^. References^5–7^ further illustrate the potential uses of many nanostructures in science and innovation.

Scientists and engineers are attracted by the thermophysical features of nanocomposites due to the widespread utilization of nanofluids in advanced technology and industrial applications. The scattering of a unique nanocomposite, although does not offer the required heat transfer performance and has no applications in industrial or technological problems. So, hybrid nanofluid is working to guarantee adequate thermal properties. According to Makishima^8^, a hybrid nanofluid is created when two or more separate nanostructures are mixed with a single conventional fluid. A possible increase in the rate of heat transfer has been shown for hybrid nanocomposites, a fascinating class of nanofluids used in a range of refrigerants, heat exchangers, thermal generators, and technological problems. Xian et al.^9^ studied the thermophysical features and durability of hybrid composites and some of their advanced characteristics. Nanofluids can be implemented into several possible purposes, such as heating systems, due to their characteristics^10,11^, pharmaceutical processes^12^, energy^13^, engine cooling^14,15^, electronics^16,17^, food and cosmetics^18^. Several experimental and numerical studies on the energy transfer and vibrational characteristics of NFs have been conducted, and the majority of these studies have shown that nanofluids can accelerate the rate of heat transfer because they have higher thermal conductivities^19–21^. On the other hand, research has demonstrated that the usage of permeable media has supplanted the dominant heat transfer^22–24^. A stable structure made up of interconnecting cavities or solid particles that are typically filled with liquid is referred to as a porous medium. Its wide contact area and tortuous shape are advantageous for accelerating heat transfer^25,26^. As a result, porous medium and nanofluids can be combined to improve heat transmission. As unique functional materials, porous media and nanofluids have important applications in improving heat transmission^27,28^. The use of both porosity and nanofluid has recently attracted a lot of interest and sparked in-depth research in this field. The area of contact between a liquid and a solid surface is increased by porous media, but heat conductivity is effectively increased by nanoparticles dispersed in nanofluid. So, it would seem that using both porous media and nanofluid might significantly boost the effectiveness of traditional thermal systems^25,29^.

In order to fulfill the requirements of both industrial and daily tasks, the researchers focused their efforts on exploring diverse and cost-effective energy sources, which encompassed sustainable energy alternatives. Nanocomposites are the key sources used for various applications including the improvement of heat transfer, energy transmission, medication, and solar disciplines. Generally, the researchers to improve the rate of energy transportation in the exchangers used active and passive strategies. Passive processes demand surface models like a rough top and elongated interface of liquids, whereas active procedures need exterior forces like a spongy surface and permanent magnets^30–32^. The nanoparticles were subjected to an electrical force, which may have an impact on the nanofluid's morphology and mobility, energy transmission is improved by an applied electric field^33,34^. The perks of such a modification involve modest design and control, and low energy consumption^35,36^.

Yang et al.^37^ employed an experimental method to determine presence of thermal waves in lagging proportion observations. They tackled a planner motion scenario by utilizing the Laplace transformation method, taking into account a tubular transmitter capable of heating an extensive volume with no apparent limit. They were used in trials to test the procedure, and it was discovered that the ratio in sand is lower than that in thin pork. Under appropriate scale uncertainty, the time delay rates for both intervals were just under 1, indicating that no thermal waves were generated. In a perforated aperture, the Sheikholeslami research^38^ modelled electrodynamic nanocomposites. In the presence of thermal radiations and an electric field, CVFEM was used to assist the modeling. Additionally, as the buoyancy forces and radiation factors climbed, the Nusselt number grows as well. Hamida et al.^39^ used the Galerkin Finite Element Method (GFEM) to show heat transfer in a duct filled with hybrid nanofluids (HNFs) operating in an electromagnetic field.

This study, which was motivated by the aforementioned studies, clarifies the hydrothermal consequences of naturally occurring, laminar, magnetically driven Ag + MgO/H_2_O hybrid nanofluid flows inside of an enclosure. The inner circular boundary remains hot while the outside round boundary is turned frigid. The complete numerical simulation is carried out using the finite element method based on the control volume (CVFEM) which provide set of information for BRT-ANN. Analyze and evaluate the expected outcomes of BRT-ANNs that were developed using the training, testing and verification datasets with the recommended solutions provider. Both nanoparticles are used in various discipline like Nanocomposites are used in anti-cancer treatments, biosensors, heat exchangers, and other applications^40,41^, whereas MgO is used in a variety of other industries, including ceramics, electronics, petroleum products, catalysts, surface coating, and many more^42^. In this work Ag + MgO/H_2_O hybrid nanofluid has been permitted to grow the thermal performance. However, Ag + MgO resulted from the highest Nusselt number $\left( {\phi = 0.05} \right)$ among all experienced cases. The results also indicated that raising the concentration of nanoparticles by 0.01, together with increasing the voltage supplied for the electric field, could improve the Nusselt number by up to 5.19% and accelerate heat transfer in the channel, respectively. For the numerical solution in this study, MATLAB (version R2019b) is utilized. Major research challenges that should be investigated during the modelling are:

- How do the velocity distributions and rate of heat transfer are affected by the Hartmann number, porosity factor, Rayleigh number and nanoparticle concentrations?
- What elements substantially change the temperature of the hybrid nanofluid?
- How can we minimize/improve the other engineering quantities of interest with the suggested hybrid nanofluid flow while proactively estimating the wall concentration?
- How are the simulation model and ANN model successfully connected?

## Description of the problem

To accomplish hybrid nanofluids, Ag and MgO are dissolved in water. In the presence of a magnetic field, the flow of a hybrid nanofluid is taken into consideration in an amorphous enclosure. In a perpendicular orientation, magnetization has been introduced. The interpretation of the sinusoidal wall pattern is

1 $$ {\raise0.7ex\hbox{$b$} \!\mathord{\left/ {\vphantom {b a}}\right.\kern-0pt} \!\lower0.7ex\hbox{$a$}} = \left( {1 - \varepsilon } \right)^{2} $$

**Figure 1:**
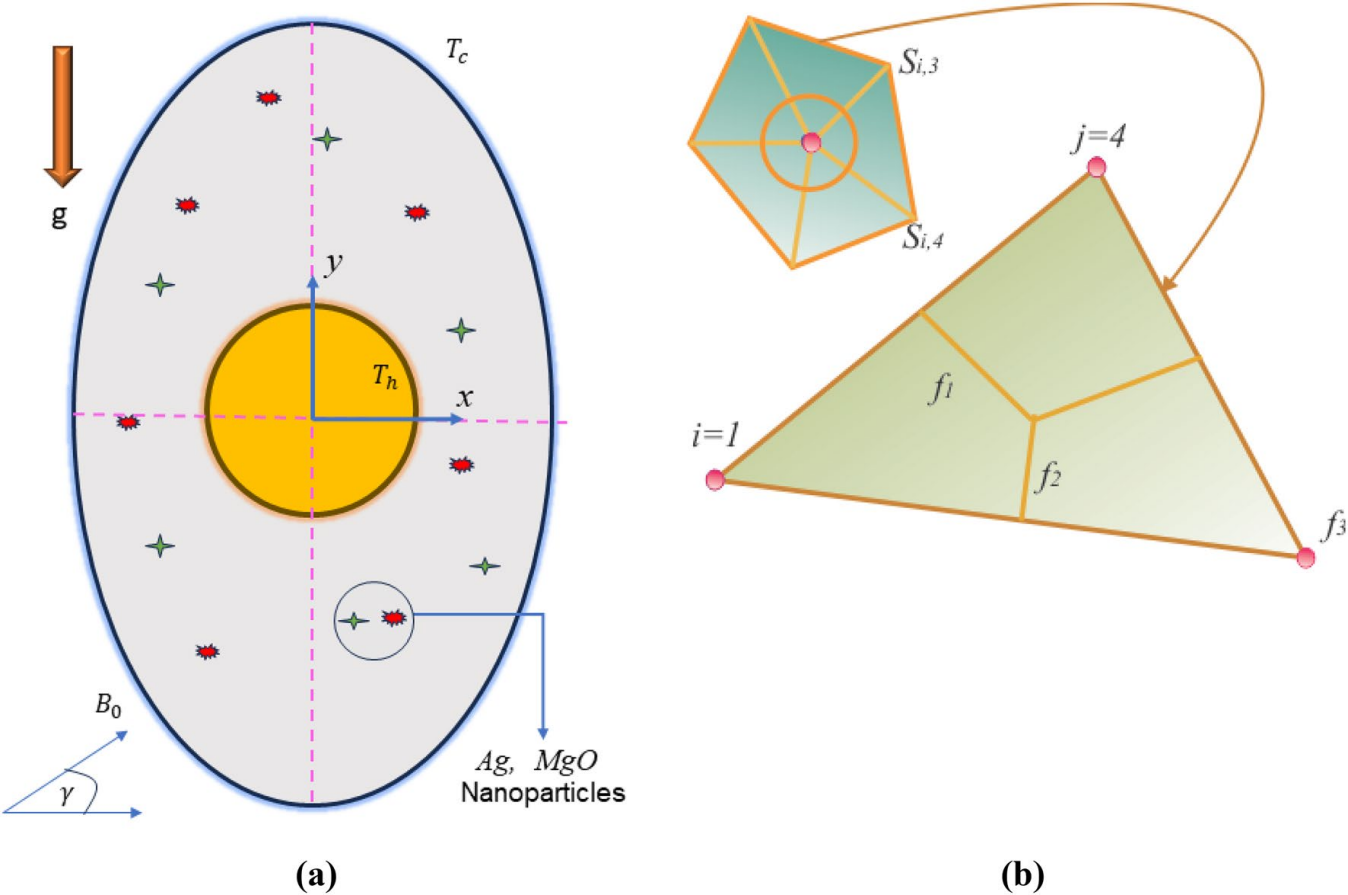
(**a**) Geometrical configuration and suppose boundary assumption using (**b**) A sampler triangular element and its associated volume control.

The boundary condition of flow and geometry is shown in Fig. 1a. The governing mathematical models for the temperature simulation using the Boussinesq-Darcy force and non-equilibrium thermal theory are as tries to follow:

2 $$ \nabla .\,\,\mathop V\limits^{ \to } = 0, $$

3 $$ \rho_{hnf} \beta_{hnf} \overrightarrow {g} \left( {\tilde{T}_{hnf} - \tilde{T}_{c} } \right) + \frac{{\mu_{hnf} }}{K} + \nabla p + \sigma_{hnf} \left( {\overrightarrow {V} \times \overrightarrow {B} } \right) = 0, $$

4 $$ \frac{{h_{hnfs} }}{{\rho_{s} \left( {cp} \right)_{s} \left( {1 - \varepsilon } \right)}}\left( {\tilde{T}_{hnf} - \tilde{T}_{s} } \right) + \frac{{k_{s} }}{{\rho_{s} \left( {cp} \right)_{s} }}\nabla^{2} \tilde{T}_{s} = 0, $$

where the relationship of hybrid nanofluids are defined as^12^:

5 $$ \left\{ \begin{gathered} \phi = \phi_{Ag} + \phi_{MgO} ,\,\,\,\,\rho_{hnf} = \left( {1 - \phi } \right)\rho_{f} + \rho_{MgO} \phi_{MgO} + \rho_{Ag} \phi_{Ag} , \hfill \\ \,\left( {\rho cp} \right)_{hnf} = \left( {1 - \phi } \right)\left( {\rho cp} \right)_{f} + \left( {\rho cp} \right)_{MgO} \phi_{MgO} + \left( {\rho cp} \right)_{Ag} \phi_{Ag} , \hfill \\ \left( {\rho \beta } \right)_{hnf} = \left( {1 - \phi } \right)\left( {\rho \beta } \right)_{f} + \left( {\rho \beta } \right)_{MgO} \phi_{MgO} + \left( {\rho \beta } \right)_{Ag} \phi_{Ag} , \hfill \\ \end{gathered} \right\} $$

6 $$ \frac{{\sigma_{hnf} }}{{\sigma_{bf} }} = \left( {1 + 3\frac{{\left( {\frac{{\sigma_{Ag} }}{{\sigma_{bf} }} - 1} \right)\phi_{Ag} }}{{\left( {1 - \frac{{\sigma_{Ag} }}{{\sigma_{bf} }}} \right)\phi_{Ag} + \left( {\frac{{\sigma_{Ag} }}{{\sigma_{bf} }} + 2} \right)}}} \right),\left. {\frac{{\sigma_{bf} }}{{\sigma_{f} }} = \left( {1 + 3\frac{{\left( {\frac{{\sigma_{MgO} }}{{\sigma_{f} }} - 1} \right)\phi_{MgO} }}{{\left( {1 - \frac{{\sigma_{MgO} }}{{\sigma_{f} }}} \right)\phi_{MgO} + \left( {\frac{{\sigma_{MgO} }}{{\sigma_{f} }} + 2} \right)}}} \right)} \right\} $$

7 $$ \left. {\frac{{k_{hnf} }}{{k_{f} }} = \left( {1 - \phi_{Ag} } \right) + \frac{{2\phi_{Ag} k_{Ag} }}{{\left( {k_{Ag} - k_{bf} } \right)}}\ln \frac{{\left( {k_{Ag} + k_{bf} } \right)}}{{2k_{bf} }},\frac{{k_{hnf} }}{{k_{f} }} = \left( {1 - \phi_{MgO} } \right) + \frac{{2\phi_{MgO} k_{MgO} }}{{\left( {k_{MgO} - k_{f} } \right)}}\ln \frac{{\left( {k_{MgO} + k_{f} } \right)}}{{2k_{f} }}.} \right\} $$

The $$k_{ef} {,}\,\,{\text{and}}\,\,\mu_{ef}$$ is

8 $$ k_{ef} = k_{sta} + k_{Bro} ,\,\,\,\mu_{ef} = \mu_{sta} + \mu_{Bro} = \mu_{sta} + \frac{{\mu_{f} }}{{\Pr_{f} }}.\frac{{k_{Bro} }}{{k_{f} }} $$

Here

9 $$ \left. \begin{gathered} k_{sta} = \left( {\left( {1 - \phi_{Ag} } \right) + \frac{{2\phi_{Ag} k_{Ag} }}{{\left( {k_{Ag} - k_{bf} } \right)}}\ln \frac{{\left( {k_{Ag} + k_{bf} } \right)}}{{2k_{bf} }}} \right)\left( {\left( {1 - \phi_{MgO} } \right) + \frac{{2\phi_{MgO} k_{MgO} }}{{\left( {k_{MgO} - k_{f} } \right)}}\ln \frac{{\left( {k_{MgO} + k_{f} } \right)}}{{2k_{f} }}} \right) \hfill \\ \mu_{sta} = \frac{{\mu_{f} }}{{\left( {1 - \phi_{Ag} } \right)^{2.5} \left( {1 - \phi_{MgO} } \right)^{2.5} }} \hfill \\ \end{gathered} \right\} $$

The Koo-Kleinstreuer-Li model for $k_{ef}$ defines as follows^43–45^:

10 $$ k_{Bro} = 5 \times 10^{4} \rho_{f} \phi \left( {\frac{{k_{b} \tilde{T}}}{{\rho_{p} d_{p} }}} \right)^{1/2} \left( {c_{p} } \right)_{f} g^{\prime}\left( {\tilde{T},d_{p} ,\phi } \right), $$

whereas the function $g^{\prime}\left( {\tilde{T},\,\,d_{p} ,\,\,\phi } \right)$ for hybrid nanofluid is identified as,

11 $$ \left. \begin{gathered} g^{\prime}\left( {\tilde{T},d_{p} ,\phi } \right) = \ln \left( {\tilde{T}} \right)\left( \begin{gathered} b_{1} + b_{2} \ln \left( {d_{p} } \right) + b_{3} \ln \left( \phi \right) + b_{4} \ln \left( {d_{p} } \right) + b_{5} \ln \left( {d_{p} } \right)^{2} + \hfill \\ b_{6} + b_{7} \ln \left( {d_{p} } \right) + b_{8} \ln \left( \phi \right) + b_{9} \ln \left( \phi \right)\ln \left( {d_{p} } \right) + b_{10} \ln \left( {d_{p} } \right)^{2} \hfill \\ \end{gathered} \right) \hfill \\ R_{f} = \frac{{d_{p} }}{{k_{p,eff} }} - \frac{{d_{p} }}{{k_{p} }} = 4 \times 10^{ - 8} km^{2} /W \hfill \\ \end{gathered} \right\} $$

Here $b_{k} ,\,\,\,\,k = [0,\,\,\,\,\,10]$ vary according to the nature of nanoparticles.

Employing the following variations^38^:

12 $$ \left. {\theta_{s} = \frac{{\left( {\tilde{T}_{s} - \tilde{T}_{c} } \right)}}{{\left( {\tilde{T}_{h} - \tilde{T}_{c} } \right)}},\,\theta_{nf} = \frac{{\left( {\tilde{T}_{nf} - \tilde{T}_{c} } \right)}}{{\left( {\tilde{T}_{h} - \tilde{T}_{c} } \right)}},v = - \frac{\partial \psi }{{\partial x}},\,\,u = \frac{\partial \psi }{{\partial y}},\,\,\Psi = \frac{\psi }{{\alpha_{nf} }},\,\,\left( {X,Y} \right) = \frac{{\left( {x,y} \right)}}{l},} \right\} $$

The dimensionless form of partial differential system is

13 $$ \begin{gathered} \frac{{\partial^{2} \Psi }}{{\partial X^{2} }} + \frac{{\partial^{2} \Psi }}{{\partial Y^{2} }} = - \frac{{L_{6} }}{{L_{5} }}Ha\left( {\frac{{\partial^{2} \Psi }}{{\partial X^{2} }}\cos^{2} \gamma + 2\frac{{\partial^{2} \Psi }}{\partial X\partial Y}\cos \gamma \sin \gamma + \frac{{\partial^{2} \Psi }}{{\partial Y^{2} }}\sin^{2} \gamma } \right)\, \hfill \\ - \frac{{L_{3} }}{{L_{4} }}\frac{{L_{2} }}{{L_{5} }}Ra\frac{{\partial \theta_{nf} }}{\partial X} - \frac{{L_{5} }}{{L_{1} }}\frac{\Pr }{{Da}}\frac{\partial \Psi }{{\partial X}}, \hfill \\ \end{gathered} $$

14 $$ \frac{{\partial^{2} \theta_{nf} }}{{\partial X^{2} }} + \frac{{\partial^{2} \theta_{nf} }}{{\partial Y^{2} }} = \frac{{\partial \theta_{nf} }}{\varepsilon \partial X}\frac{\partial \Psi }{{\partial Y}} - \frac{{Nhs\left( {\theta_{s} - \theta_{nf} } \right)}}{\varepsilon } - \frac{{\partial \theta_{nf} }}{\varepsilon \partial Y}\frac{\partial \Psi }{{\partial X}} $$

15 $$ \frac{{\partial^{2} \theta_{s} }}{{\partial X^{2} }} + \frac{{\partial^{2} \theta_{s} }}{{\partial Y^{2} }} = - \frac{{Nhs\left( {\theta_{nf} - \theta_{s} } \right)}}{\varepsilon } $$

where the non-dimensional factors are:

16 $$ \left. \begin{gathered} L_{1} = \frac{{\rho_{hnf} }}{{\rho_{f} }},\,\,\,L_{2} = \frac{{\rho_{hnf} \left( {cp} \right)_{hnf} }}{{\rho_{f} \left( {cp} \right)_{f} }},\,\,\,L_{3} = \frac{{\rho_{hnf} \left( \beta \right)_{hnf} }}{{\rho_{f} \left( \beta \right)_{f} }},\,\,L_{4} = \frac{{k_{hnf} }}{{k_{f} }},L_{5} = \frac{{\mu_{hnf} }}{{\mu_{f} }},\,\,\,\,L_{6} = \frac{{\sigma_{hnf} }}{{\sigma_{f} }}, \hfill \\ Ra = \frac{{gK\rho_{f} \left( \beta \right)_{f} \Delta \tilde{T}}}{{\mu_{f} \alpha_{f} }},\,\,Nhs = \frac{{h_{hnfs} l^{2} }}{{k_{hnf} }},\,\,\,\delta_{s} = \frac{{k_{hnf} }}{{k_{f} \left( {1 - \varepsilon } \right)}},\,\,\,\,\,Ha = \frac{{KB_{0}^{2} \sigma_{f} }}{{\mu_{f} }},Da = \frac{k}{{l^{2} }} \hfill \\ \end{gathered} \right\} $$

Given that the inner side is presumed to be heated, the boundary requirements are as follows:

17 $$ \left. \begin{gathered} {\text{On}}\,\,{\text{all}}\,\,{\text{walls}} = \Psi = 0, \hfill \\ {\text{On}}\,\,{\text{the}}\,\,{\text{outer}}\,\,\,{\text{wall}} = \theta_{s} = 0,\,\,\,\theta_{nf} = 0, \hfill \\ {\text{On}}\,\,{\text{the}}\,\,{\text{inner}}\,\,{\text{wall}}\theta_{s} = 1,\,\,\,\,\theta_{nf} = 1, \hfill \\ \end{gathered} \right\} $$

Here the local and average Nusselt number, when the wall is cold:

18 $$ Nu_{loc} = \frac{{k_{nf} }}{{k_{f} }}\frac{{\partial \theta_{nf} }}{\partial r},\,\,\,\,\,Nu_{ave} = 0.5\pi \int\limits_{0}^{2\pi } {Nu_{loc} dr} $$

### CVFEM modelling and grid test

**Figure 2:**
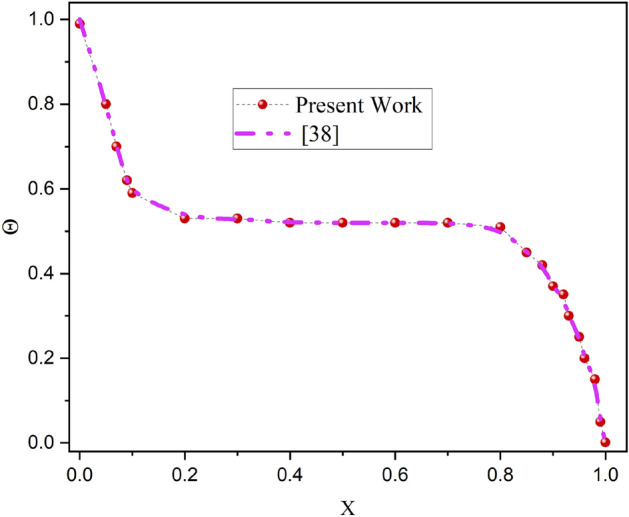
Validation of current outcomes with previous work ^38^.

**Figure 3:**
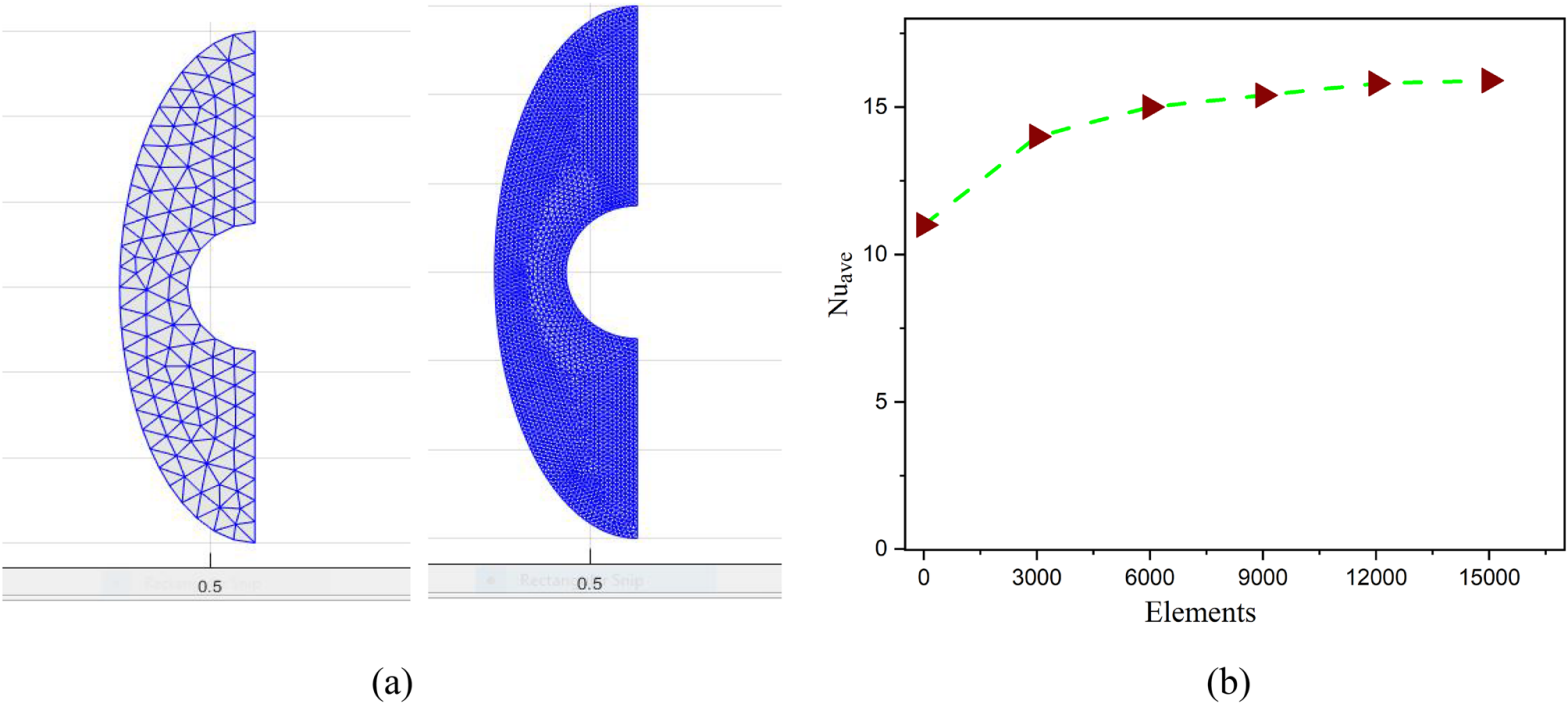
(**a**) The grid presentation of the proposed model, (**b**) The grid test profile.

The suggested modeling approach shown in Eqs. (11–14) has been numerically solved using an advanced CVFEM procedure. The discrete form of partial differential equation is typically displayed in space using a globally determined coordinate system in the finite element approach. The proposed method uses hexahedral elements to discretize the physical domain. Elements are separated into smaller control volumes in the new destination. For excellent outcomes, it is important to consider the ideal grid design. The quantity of grids has a significant impact on the overall computational complexity and the reliability of model analyzed data. Adopting narrow grids, which result in significant discretization mistakes, causes inaccurate research outcomes. The round-off error, however, could grow to be much bigger than the truncation error if the grid is too narrow, which would produce less reliable results^6^. Therefore, choosing the appropriate quantity of grids is important^7^. In several CFD studies, the ideal grid size was determined through grid independence analysis. (Fig. 2) demonstrates the comparison between the current study and earlier available research showing a strong level of agreement which present the originality of the present research work. The grid independence test can identify which grid configuration yields the best overall numerical results with the least quantity of grids by analyzing the mathematical data achieved with various grid dimensions and intensities. The proper mesh has been utilized in each scenario and the solution range is not just evaluated on the grid size in CVFEM code. For perfect precision in the case of high grids, a more sophisticated computer has been employed to locate the solution. Figure 3a, illustrates the grid presentation of the suggested model. In order to meet the requirements of the grid sensitivity test, 15920 components are chosen for this mathematical calculation, as shown in Fig. 3b.

For the outcomes of the model expression, the MATLAB software's "CVFEM" function implements a numerical technique. The neural network is developed employing data source that considers variants connected to the proposed nanofluid movement mechanism in the regions 0 and 4. The CVFEM strategy, which utilizes configuration settings for iterations, consistency objective, and acceptance rate for solving prevalent mathematical equations, is adapted in MATLAB software to support the proposed neural network approach.

### Designation of artificial neural networks modeling

**Figure 4:**
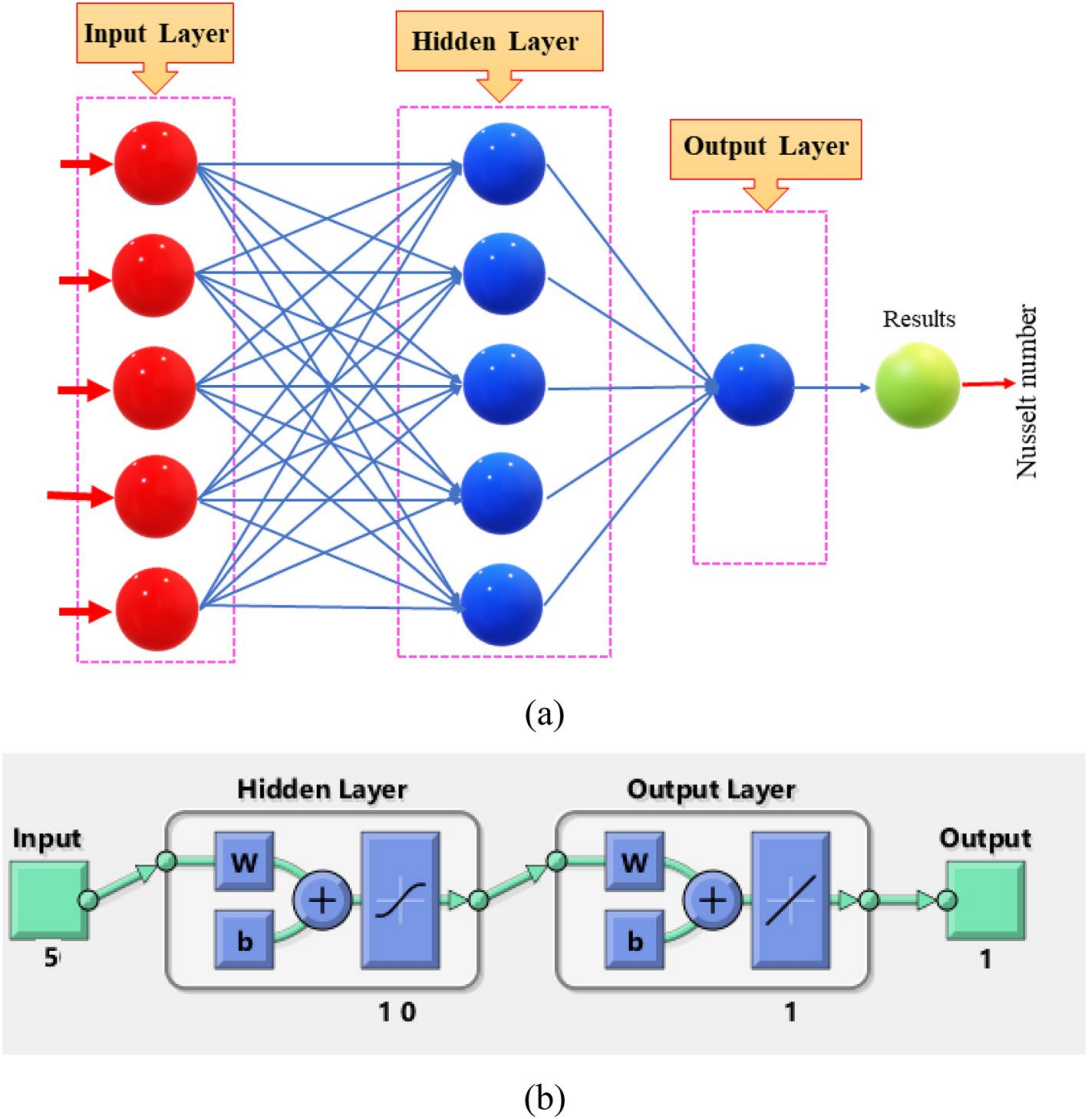
(**a**) A model configuration for singular neural networking, **(b)** Design of a planned neural network.

The NF-tool (neural network fitting tool) is then used on a sequence similar to that described in ^46,47^. A single neural network model is presented in Fig. 4a. The suggested network's structure is presented in Fig. 4b and the BRT-ANN is constructed employing MATLAB's NF tool with the appropriate settings of unseen neurons, testing datasets, training datasets, and validation datasets. Software is used to train a neural network's weight function via Bayesian Regularization backpropagation. To achieve optimization, the suggested BRT-ANN incorporates a multi-layer neural network structure with Bayesian Regularization backpropagation. The BRT-ANN procedure was implemented to obtain the results of a hybrid nanofluid flow in a porous cavity system using the NF-tool with 5 neurons in the hidden layer by varying $Da,\,Ra,\,Ha$ and $\delta s$ for various values. The datasets for learning, verification and evaluation were allocated 70%, 15%, and 15%, respectively. Tan-Sig formulation was utilized for transmission in ANN models with hidden nodes along with Purelin function was used for output nodes^48^. The transfer function can be changed in the manner described below:

19 $$ f(x) = \frac{1}{1 + \exp ( - x)}, $$

20 $$ {\text{Also}},\;\;\;{\text{Purelin}}(x) = x. $$

Evaluating the predictive capability of ANN models is significant after the construction of ANN models and the obtaining of predicted results. The predictive performance of ANN models has been evaluated using the MSE (mean squared error), R (coefficient of determination) and error rate metrics. Below is a representation of the algorithms used to estimate the system performance^49,50^.

21 $$ \left. \begin{gathered} {\text{Mean}}\,{\text{Square}}\,{\text{Error}} = \frac{1}{\hbar }\sum\limits_{k = 1}^{\hbar } {\left( {{\rm X}_{\exp (k)} - {\rm X}_{ANN(k)} } \right)^{2} } , \hfill \\ {\text{R}} = \left( {\frac{{\sum\limits_{k = 1}^{\hbar } {\left( {{\rm X}_{\exp (k)} } \right)^{2} } - \sum\limits_{k = 1}^{\hbar } {\left( {{\rm X}_{\exp (k)} - {\rm X}_{ANN(k)} } \right)^{2} } }}{{\sum\limits_{k = 1}^{\hbar } {\left( {{\rm X}_{\exp (k)} } \right)^{2} } }}} \right)^{{{\raise0.7ex\hbox{$1$} \!\mathord{\left/ {\vphantom {1 2}}\right.\kern-0pt} \!\lower0.7ex\hbox{$2$}}}} , \hfill \\ {\text{Percentage Error rate = 100}} \times \left( {\frac{{{\rm X}_{\exp } - {\rm X}_{ANN} }}{{{\rm X}_{\exp } }}} \right). \hfill \\ \end{gathered} \right\} $$

## Results and discussion

**Figure 5:**
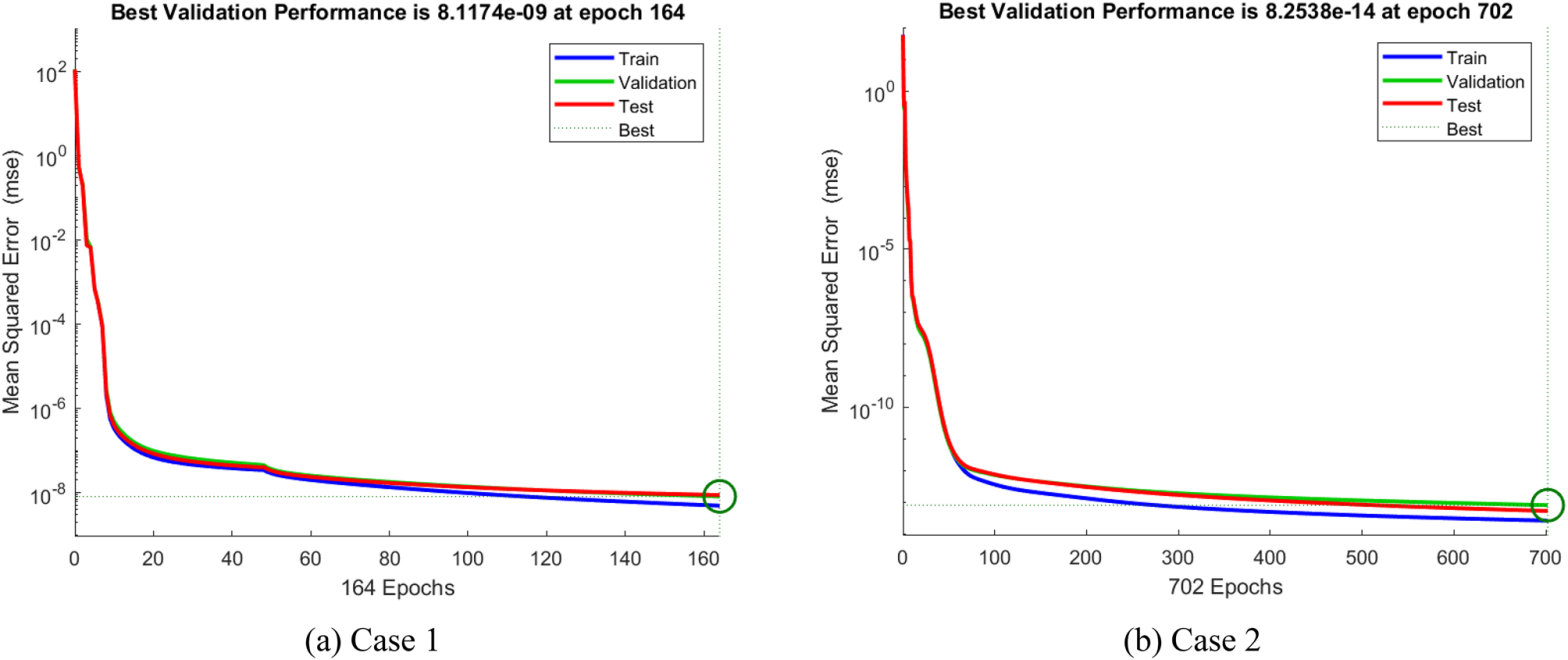
Plots of mean square error results for Porous Cavity BRT-ANN model.

**Figure 6:**
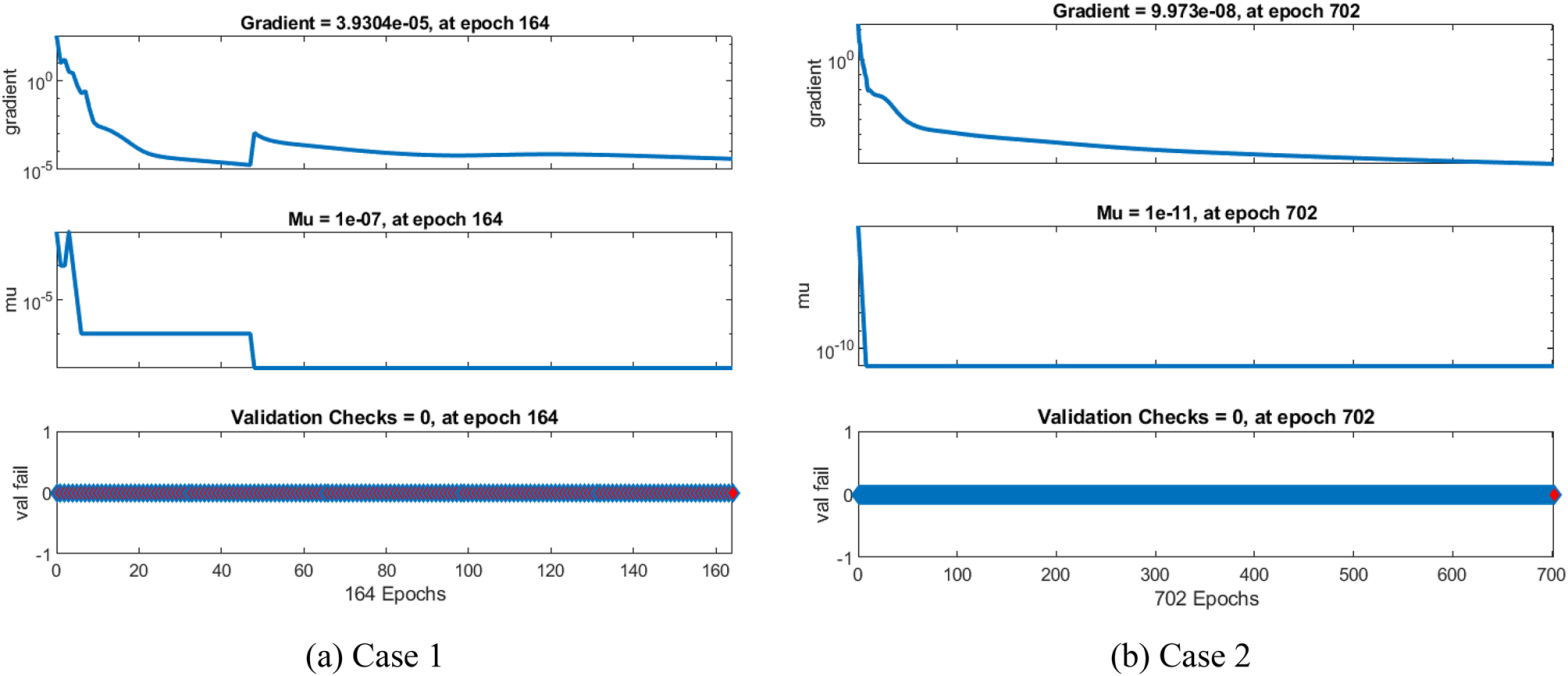
The designed transition state for BRT-ANN model.

**Figure 7:**
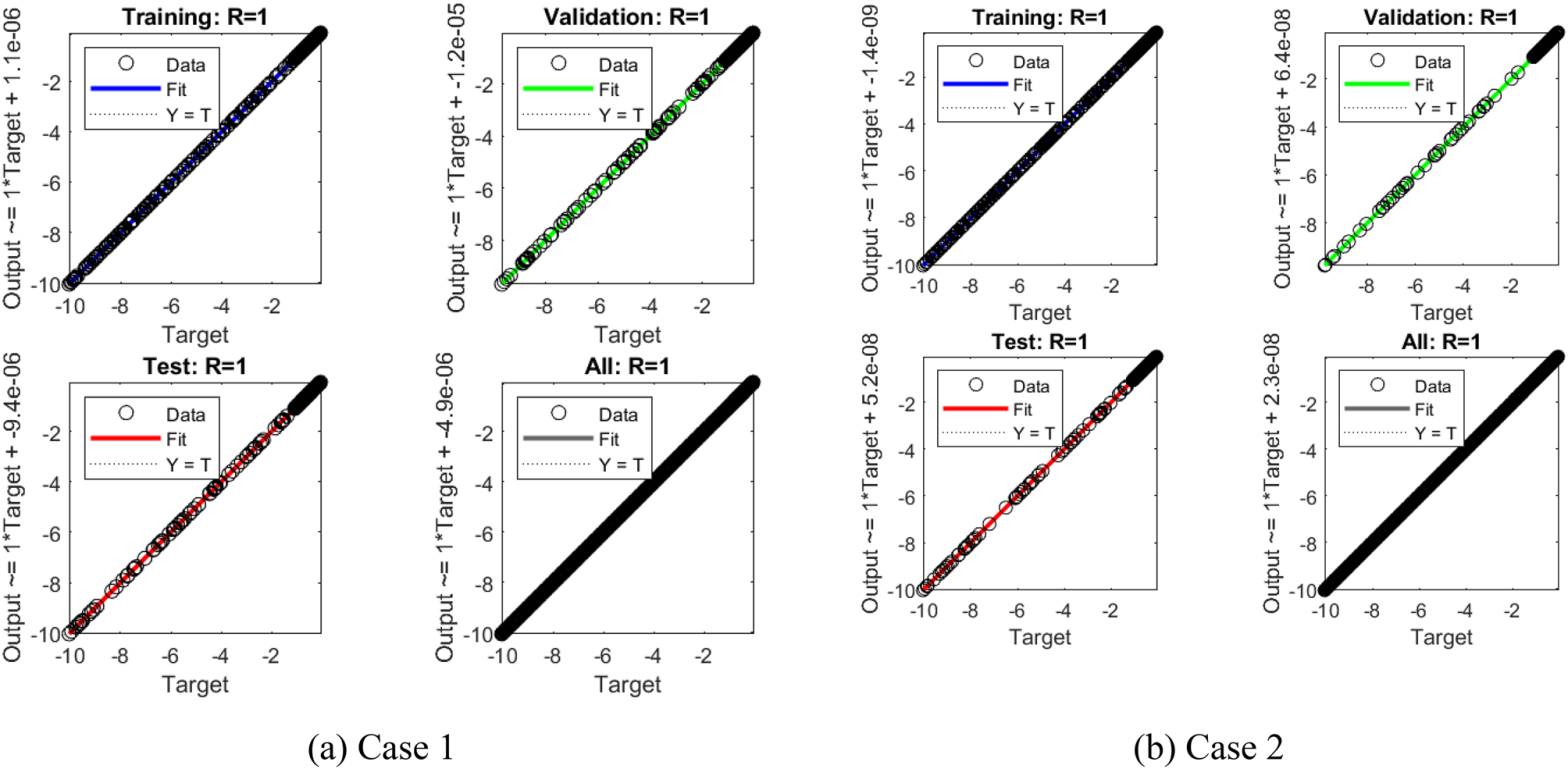
The designed plots of Regression for BRT-ANN model.

**Figure 8:**
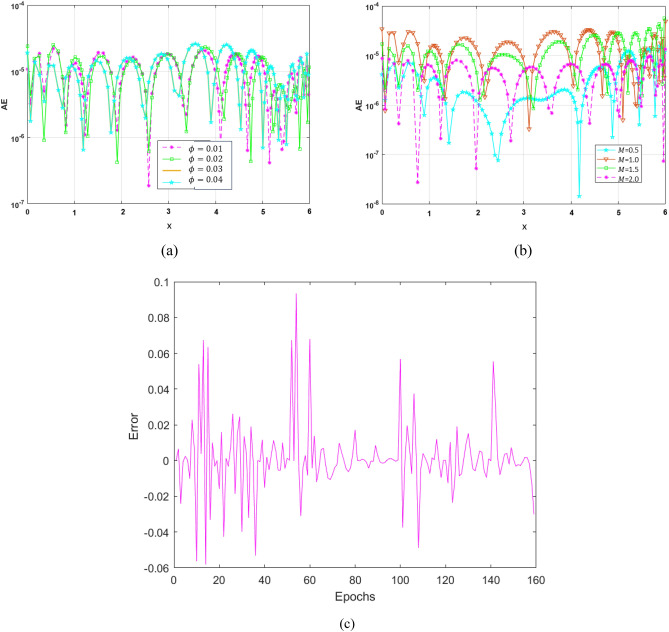
Plots of the thermal profile produced by AE for varying (**a**) $\phi$, (**b**) $M$ and (**c**) error analysis with various epochs.

**Figure 9:**
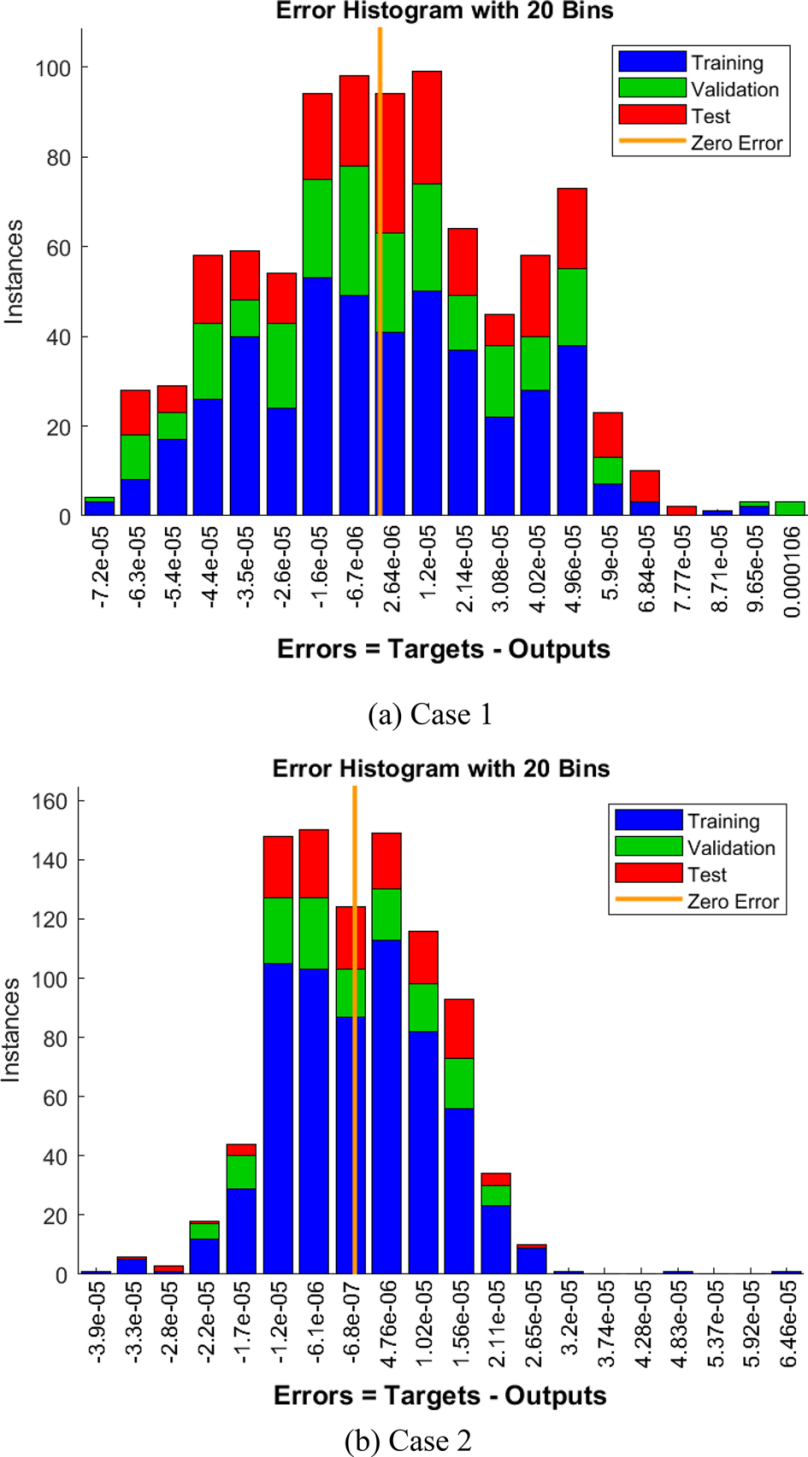
The error Histogram for designed BRT-ANN model.

A non-equilibrium simulation has been used to demonstrate how a magnetic field affects the mobility of hybrid nanofluids inside a perforated enclosure. For the high grid formulation, the computational technique (CVFEM) was employed. The results examine the impact of modifying the physical parameters like Rayleigh number, porosity factor and the Hartmann number. The thermophysical data of nanocomposites are presented in Table 1. The profiles of velocity as well as their AE (absolute error) analysis graphs for two cases are shown in Figs. 5 and 6 for the BRT-ANN findings of the present model for two cases. The geometrical configuration and suppose boundary assumption and a sampler triangular element and its associated volume control are presented in Fig. 1a,b. Figure 2 and Table 2 illustrate how the results of the current study and previous research^35^ and^36^ have been validated. Table 3 displays the collected data, which illustrate that the $Nu$ variations dropped as the mesh quality grew, leading us to the conclusion that the highest grade, extra fine mesh guaranteed correct results. The numerical changes in $Nu_{ave}$ against $Ha$ for the various values of $\phi_{Ag}$ and $\phi_{MgO}$ are shown in Table 4. The results of BRT-ANN for the flow model to solving various cases are presented in Table 5. This outcome presents that the attained finding is comparable to the available work considering common parameters. Figure 3a is the representation of the suggested model for the number of grids in smaller and higher while Fig. 3b signifies the grid test profile. The graphical representation in Fig. 5a,b depicts the training performance of BRT-ANN models of two slected cases. Initially, MSE (mean squared error) magnitude are greater, but as the quantity of train epochs improves, they decrease gradually. That is possible to see the convergence of the shapes generated via statistics from the BRT-ANN testing, verification and trained processes and the best line is indicated by dotted lines at epochs (164 and 702). Once theBRT-ANN achieves the value of lowermost mean square error at these epochs, signifying the conclusion of the training mood later several repetitions of epochs, the model's training is deemed to be complete.Such strategy denotes that the superior concert training stage of ANN simulation has been successfully finalized. Figure 6a,b graphically illustrates the training states of BRT-ANN models, including the gradient coefficient, mu and validation checks for two cases. The graphs depict how the gradient coefficient varies with an increasing number of epochs, and demonstrate that the regression results for the final gradient are almost zero. Additionally, the graphs display fluctuations in the values of mu, that imitate changes in the BRT-ANN weights. The results represent that as the quantity of epochs rises, then the numbers of smallest gradient coefficient keep falling, eventually resulting in the adoption of the excellent and suitable levelsof errors from BRT-ANN models after several testing process. These outcomes show that the ANNs' training operations were successfully finished. The training stages of BRT-ANN models are depicted in Fig. 7a,b, where the x-axis represents the target values and the y-axis displays the BRT-ANN predictions (output) for two cases. The solid compatibility (fit) line exhibits the graphical representation of the data points collected during the training process. The R value denotes the magnitude of the relationship between the target and output values, and the solid line shows the linear regression line that fits the target and output values. The computation of the regression analysis resulted in an R = 1, a precise linear correlation between the output and the targeted values.These findings demonstrate that the BRT-ANN models have effectively completed the trainings mood with minimal levels of error. Figure 8a illustrates how the velocity of the nanofluid decreases with an increase in $\phi_{1} ,\,\phi_{2}$ due to an improvement in the nanoparticle volume fractionSuch findings suggest that the BRT-ANN simulation magnificently ended the training stage with very little error. The impact of the magnetic component on the resulting nanofluid flow is depicted in Fig. 8b. Figure 8c the error analysis for distincet epochs. Actually, the graphs show that $M$ has a diminishing impact on the dynamical profiles connected to nanofluid velocity. It is significant to analyze the error histogram to measure the efficiency of BRT-ANN models. Figure 9a,b provides a graphical representation of the predicted errors from multilayer perceptron network models by subtracting the outputs from the targets for two selected cases. The visualizations of the error histograms show that the errors from each stage of the BRT-ANN model are relatively small. It is clear that errors build up as they approach the zero-error line. As compared to the baseline error with surrounding errors, the average error bin for the developed BRT-ANN models is $6.8 \times 10^{ - 7} ,\,\,2.64 \times 10^{ - 6}$ respectively.

**Table 1:** Ag and MgO nanoparticles thermophysical characteristics ^21,22^.

Properties	Water	Ag	MgO
Density $\left( {{\uprho } = {\text{kg/m}}^{{3}} } \right)$	997.10	10500	3580
Heat capacity $\left( {{\text{C}}_{{\text{p}}} {\text{ = j/kgk}}} \right)$	04179	00235	879
Thermal conductivity $\left( {{\text{k = W/m}} \cdot {\text{k}}} \right)$	0.6130	00429	30
Thermal expansion $\left( {{\upbeta } \times 10^{5} = K^{ - 1} } \right)$	00021	$5.4 \times 10^{ - 5}$	$33.6 \times 10^{ - 6}$
Electrical conductivity $\left( {\sigma = \frac{s}{m}} \right)$	$5.5 \times 10^{ - 6}$	$8.1 \times 10^{ - 4}$	$8 \times 10^{ - 4}$
$\left( {\alpha = \frac{{m^{2} }}{s}} \right)$	$1.47 \times 10^{ - 7}$	$147 \times 10^{ - 3}$	$95.3 \times 10^{ - 7}$

**Table 2:** Evaluation of present and reported outcomes.

$Ha$	$Nu_{ave}$ ^38^	$Nu_{ave}$ ^35^	$Nu_{ave}$ [Present]
1	2.5745	2.57959	2.5736
5	2.3985	2.3998	2.3976
10	2.2569	2.25793	2.2570
15	1.7786	1.7797	1.7791

**Table 3:** Computational analysis of grid independence test**.**

Mesh quality	$Nu$	$Nu$ deviation (%)
0.8220	3.6	12.14
0.8825	4.24	7.25
0.8912	4.55	0.62
0.9002	4.72	–

**Table 4:** Effect of $Ha$ on $Nu_{ave}$ for various values of $\phi_{Ag}$ and $\phi_{MgO}$**.**

$Ha$	$\phi_{Ag}$	$\phi_{MgO}$	$\phi$	$Nu_{ave}$
10	0.01	0.01	0.02	23.3451
20	–	–	–	8.6732
30	–	–	–	4.4703
10	0.03	–	0.06	22.4527
20	–	–	–	8.2319
30	–	–	–	4.3226
10	0.01	0.03	0.06	22.03811
20	–	–	–	8.1910
30	–	–	–	4.0137
10	0.04	–	0.08	21.1830
20	–	–	–	5.7352
30	–	–	–	3.9071

**Table 5:** The outcomes of BRT-ANN for the flow model.

Case	Epoch	MSE			Performance	Gradient	Time	Mu
		Train set	Validating set	Test set				
1	164	1.272E−9	2.013E−8	3.221E−8	8.1174E−09	5.33E−06	0:00:21	1.00E−07
2	702	1.328E−7	1.280E−9	2.520E−9	8.2538E−11	2.51E−05	0:00:27	1.00E−11
3	125	2.710E−8	3.517E−8	2.291E−9	4.2073E−08	4.26E−04	0:00:10	1.00E−09

**Figure 10:**
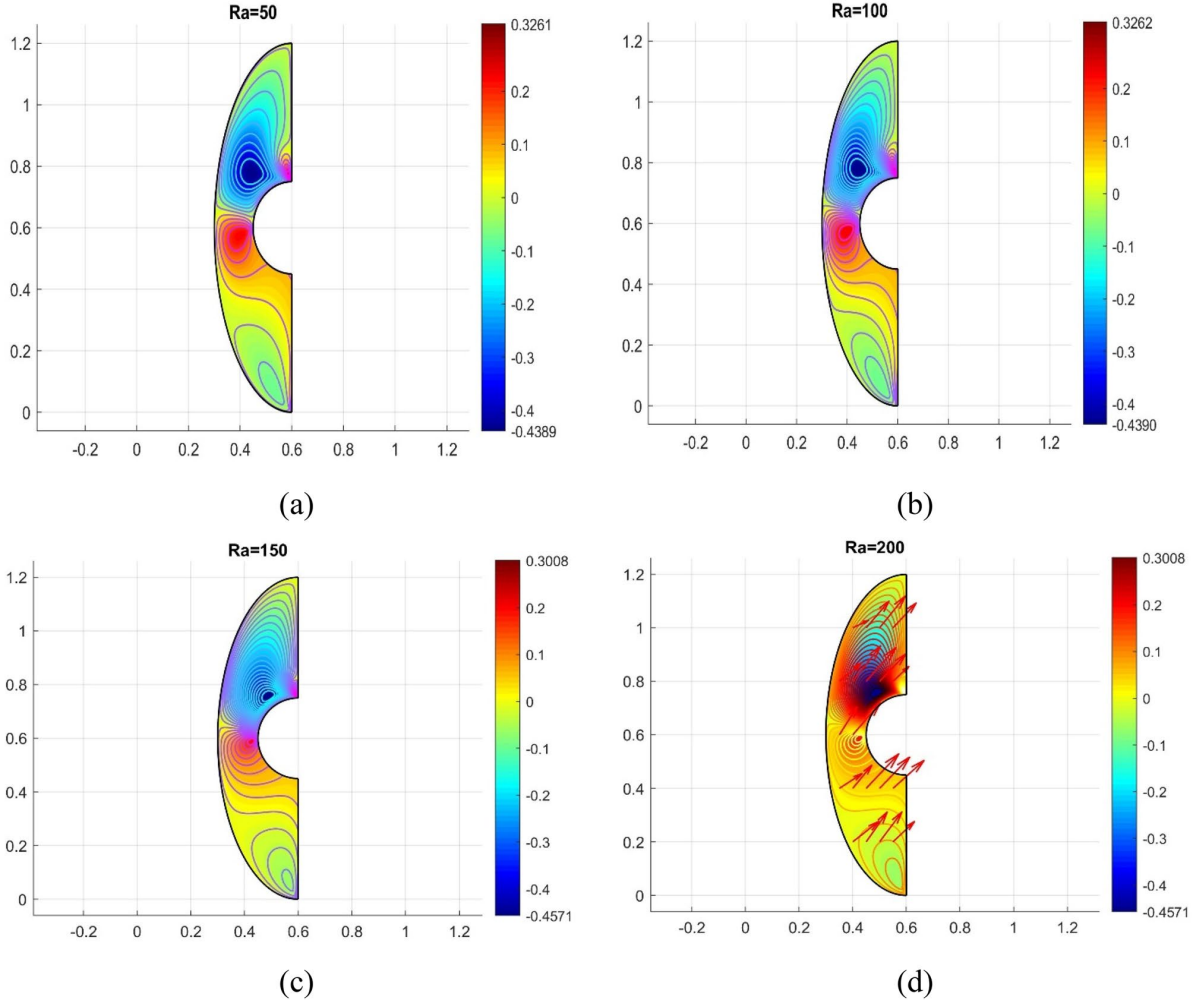
Variation in different Rayleigh numbers for velocity profile.

**Figure 11:**
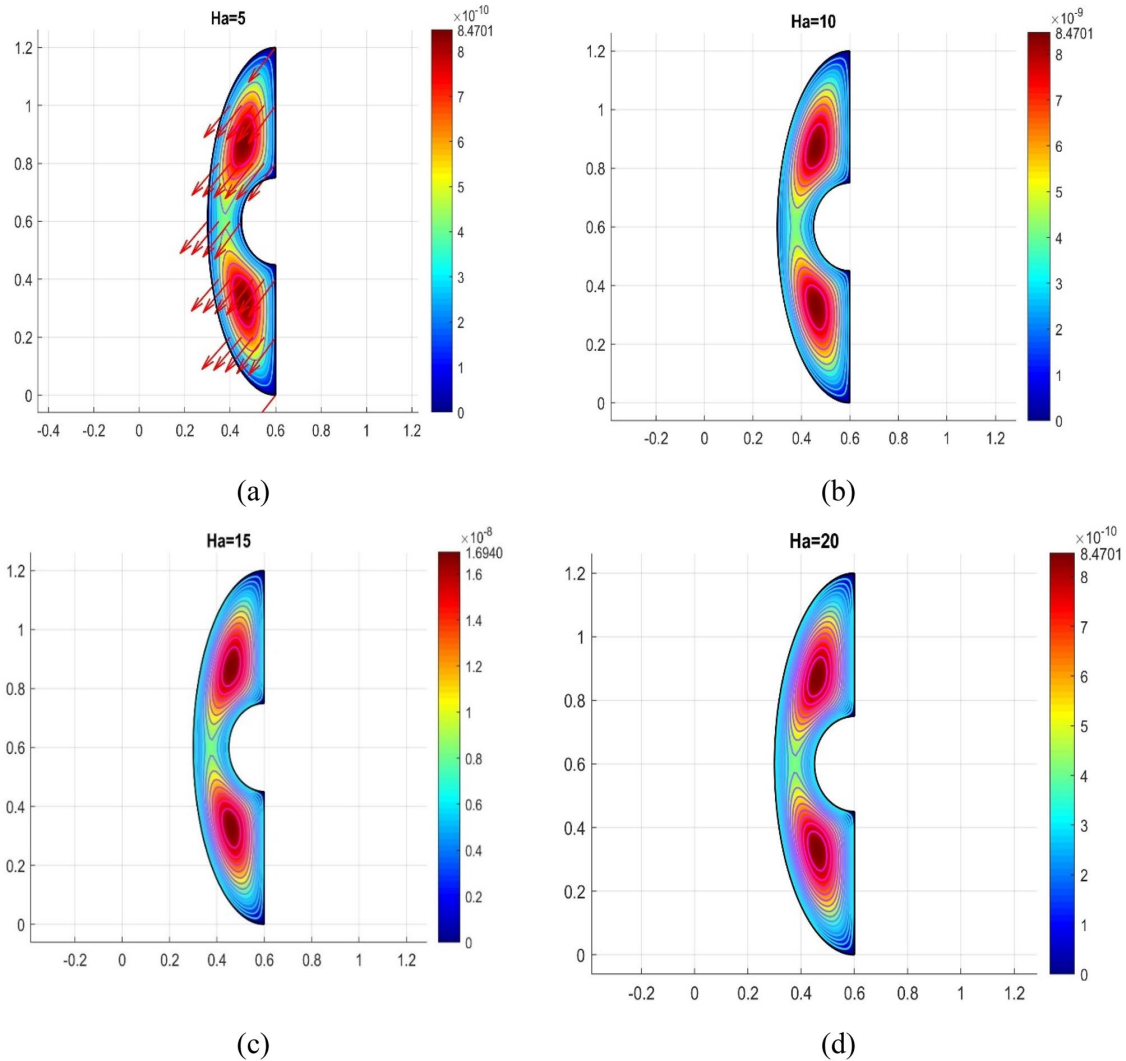
Variation in different Hartmann numbers for velocity profile.

**Figure 12:**
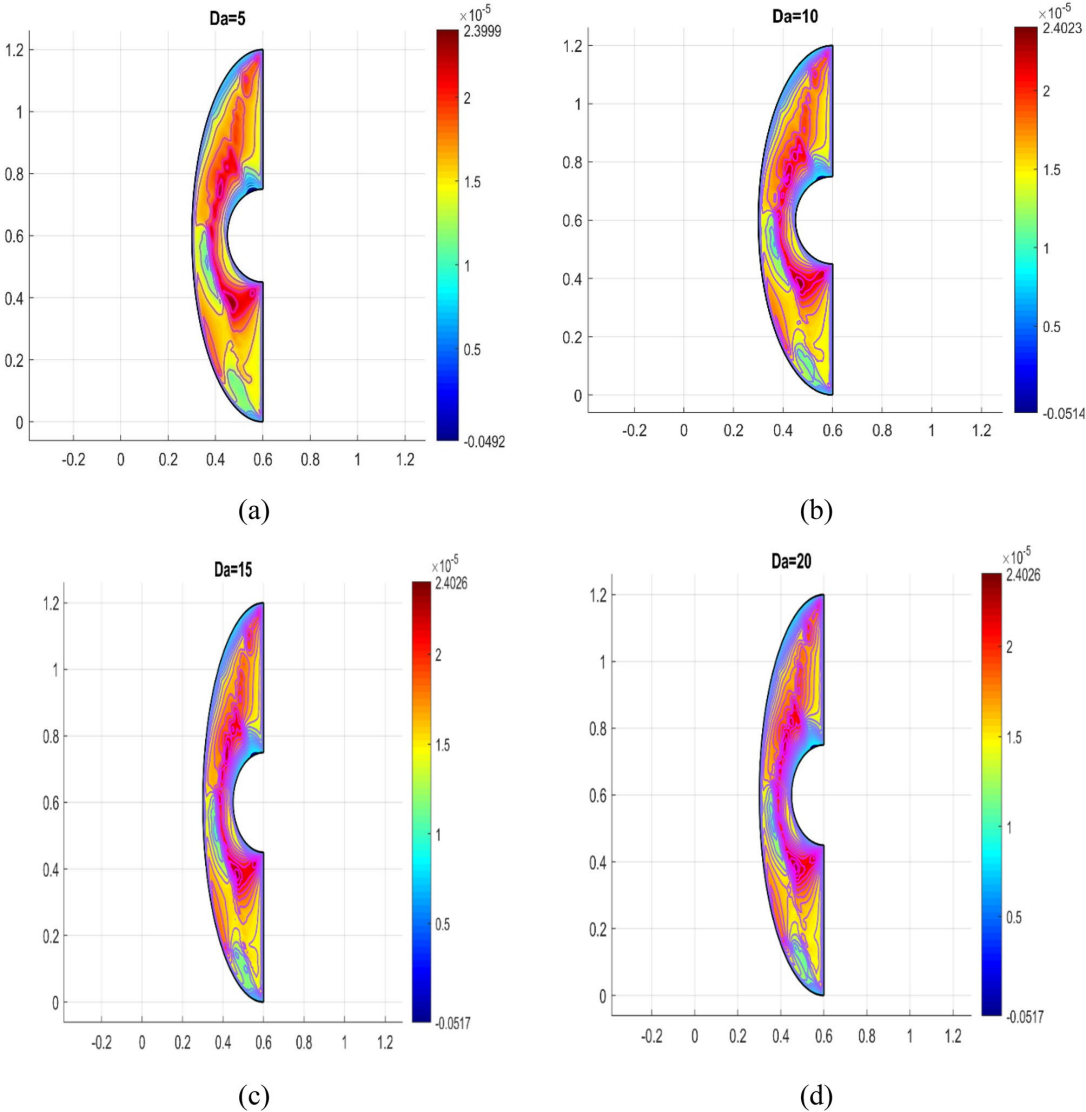
Variation in different porous parameters for velocity profile.

**Figure 13:**
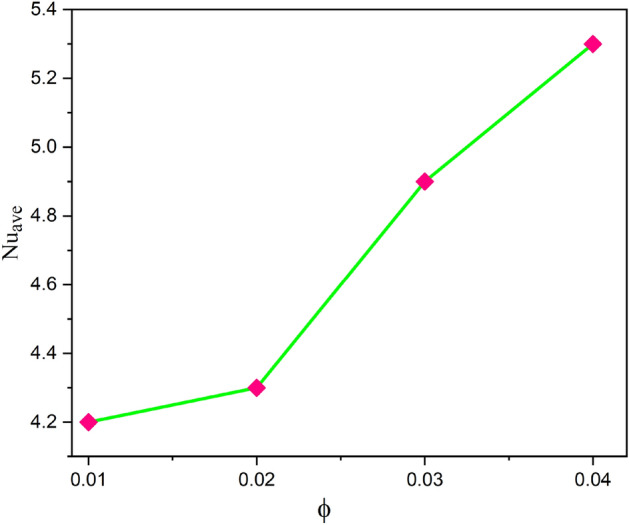
Variations of $Nu_{ave}$ with $\phi$ when $Ra = 110,Da = 5,Ha = 10$.

**Figure 14:**
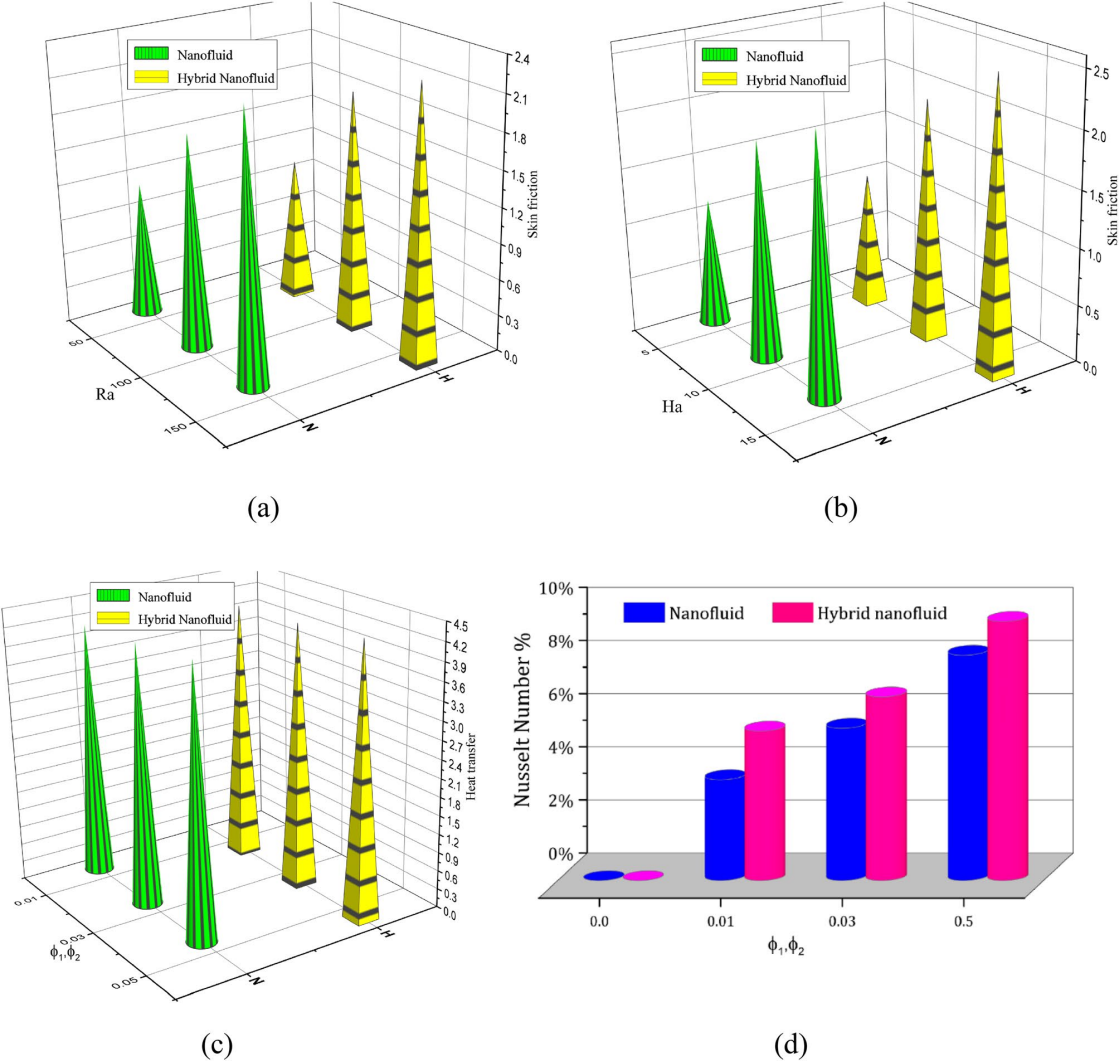
Variation in Skin friction versus (**a**) $Ra$, (**b**) $Ha$, (**c**) Nusselt number versus volume fraction and (**d**) improvement in the percentage of heat transmission.

The influence of various model flow parameters such as $Ra$ and $Ha$ on the velocity filed in the axially and rotational magnitudes were shown in Figs. 10 and 11. The fluid flow pattern is defined by the Rayleigh number in regard to buoyancy-driven flow, commonly known as free convection. Since the conduction stage is steady and the convectional motion of fluid is minimal for low Rayleigh numbers, the energy trajectories have the same pattern. The thermal boundary layer on the surface of the inner wall thins as the $Ra$ rises gradually ($Ra$ = 50, 100, 150, and 200), as revealed in Fig. 10 a, b, c and d, suggesting that convectional is more important for heat transfer at these maximum amounts. Also, the topmost portion of the internal spherical wall is starting to develop a cloud. A strong cloud is pushing the flow forcefully up against the top of the box at this point. The center of the primary vortices also keeps rising as the convection velocity rises. Thus, the Lorentz force, together with a rise in Rayleigh number and a drop in Hartmann number, confines the nanofluid movement as shown in Fig. 11a, b, c and d. In addition to the fact that conduction in the porous medium is significantly stronger than natural convection, the isotherms on permeable surfaces become more contorted as the flow quality improves. This is because there is more naturally occurring convection in the free flow. As a result, conductions and natural convection have replaced heat transfer as the primary means of controlling energy transmission in porous surfaces. As shown in Fig. 11a, b, c and d the oppositional force can be used to block the passage of liquid more effectively as the amount of Hartmann number ($Ha$ = 5,10,15, and 20) rises. Therefore, a drop in average temperature at the porous medium's interface results from the values of $Ra$ enhancement. Figure 12a, b, c and d illustrates how the thermal cloud decreases when the quantity of ($Da$ = 5, 10, 15 and 20) rises. The boosting magnitude of porosity parameter improve the resistive forces to decline the fluid flow. The variations of $Nu_{ave}$ with various values of $\phi$ are shown in Fig. 13. The $Ra$ enhances the drag force for the high magnitude and such influence is extra apparent in presence of hybrid nanofluid as demonstrated in Fig. 14a. Also, the increasing strengths of the $Ha$ improve the drag force as shown in Fig. 14b. The rate of energy transportation enhances due to the increase in solid nanostructure as presented in Fig. 14c. The nanoparticle volume fraction increase demonstrates that hybrid nanofluids are superior in the enhancement of energy transmission as presented in Fig. 14d. The percentage wise improvement demonstrates that hybrid nanocomposites are more successful in growing the energy transmission rate.

## Conclusion

The hybrid nanofluids flow considering Ag, and MgO nanoparticles are used for the augmentation of energy transformation in a lid-driven permeable enclosure and are exhibited utilizing CVFEM strength of AI based computing with BRT (Bayesian Regularization technique) of artificial neural networks. Due to its superiority over conventional mathematical models and their newfound success, ANNs are one of the engineering tools that are widely employed by many scientists. Consequences are conveyed for different magnitudes of $Ha$, $Da,\,\,Ra$ and $\phi$. The following significant physical inferences can be extracted from the comprehensive computation studies carried out by CVFEM and BRT-ANNs for the system:

- The observations show that the velocity of the fluids (Ag + MgO/Water) greatly decreases toward the middle of vessel due to an enhancement in the magnitude of flow parameters $Ra,Ha$ and $\phi$.
- The temperature distribution for hybrid nanofluid seems to be consistently greater for traditional fluids.
- The cavity's design has a small impact on the flow and heat transport mechanisms. The rate of energy transmission is amplified in a cavity with sharper edges.
- The magnetic field's influence gradually slows down the rate of energy transmission. The performance of hybrid nanocomposites as an energy transmission medium in the cavity is not significantly impacted through the inclination angle of the magnetic field.
- Thermal efficiency of hybrid nanofluids massively increase with a little increment in volume fraction.
- The MSE value, R value and average error rate for the ANN design model to predict the Nusselt number have been calculated as $1.13 \times 10^{ - 5}$, 1 and 0.02%, respectively.
- In the upcoming analysis, research in the presented BRT-ANNs based single network might be performed to model the estimates of all benchmark results determined by the CVFEM procedure base numerical outcomes of various fluid models.

## Data Availability

All the data is given within the manuscript.

## List of symbols

$$u$$_,_$$v$$: Velocities components $$\left( {{\text{ms}}^{{ - 1}} } \right)$$
$B$: Magnetic field strength $\left( {{\text{NmA}}^{{ - 1}} } \right)$
$$a,\,b$$: Constants
$$T$$: Fluid temperature $$\left( {\text{K}} \right)$$
$$p$$: Pressure distribution
$$K$$: Permeability
$X,Y$: Non-dimensional coordinates
$$Da$$: Pressure distribution
$$Ha$$: Hartmann number
$$Ra$$: Reyleigh number
$${\rm N}u$$: Nusselt number
$C_{p}$: Specific heat of base fluid $\left( {\text{J/kgK}} \right)$
$$k$$: Thermal conductivity ($${\text{Wm}}^{{ - 1}} {\text{K}}^{{ - 1}}$$)
$$\Pr$$: Prandtl number
$Nhs$: Interface heat transfer parameter
$\delta_{s}$: Thermal conductivity ratio

## Abbreviations

CVFEM: Control volume finite element method
BRT-ANN: Bayesian regularization technique of artificial neural network
AE: Absolute error

## Greek symbols

$$\mu$$: Dynamic viscosity $$\left( {{\text{mPa}}} \right)$$
$$\rho$$: Density $$\left( {{\text{Kgm}}^{{{\text{3}}}} } \right)$$
$$\varepsilon$$: Porosity
$$\beta$$: Thermal expansion
$$\xi$$: Similarity variable
$\phi$: Nanoparticle volume fraction
$\theta$: Dimensional heat profiles
$$\sigma$$: Electrical conductivity
$$\psi$$: Stream function
$$\gamma$$: Inclination

## Subscripts

*p*: Particles
*hnf*: Hybrid nanofluid
*nf*: Nanofluid
*s*: Solid nanoparticles
*f*: Base fluid
